# Evaluation of potential role of Atp5g3 in modulating alcohol preference and obesity

**DOI:** 10.1186/1471-2105-13-S12-A17

**Published:** 2012-07-31

**Authors:** Yue Huang, Lishi Wang, Robert W Willliams, Weikuan Gu, Yan Jiao

**Affiliations:** 1 Department of Orthopedic Surgery, University of Tennessee Health Science Center, Memphis, TN 38163, USA; 2 Department of Anatomy and Neurobiology, University of Tennessee Health Science Center, Memphis, TN 38163, USA

## Background

Mitochondrial ATP synthase, subunit c, isoform 3 (*Atp5g3*), encodes subunit 9 of a multisubunit enzyme that catalyzes the synthesis of ATP during oxidative phosphorylation. Each ATP synthase complex has multiple copies of subunit 9 in its transmembrane portion (Fo). While much is known about the molecular mechanisms and function of this complex in the mitochondrial membrane, the impact of variants in this complex and its connection to other pathways and human diseases and disorders is unknown.

## Materials and methods

Using extensive phenotype and gene expression data sets in GeneNetwork, we generated correlations between variation in the expression of *Atp5g3* in three tissues (hippocampus, cerebellum, and liver) and traits that are related to alcoholism and metabolism/obesity.

## Results

With limited numbers of strains, current data in GeneNetwork suggested potential associations between expression levels of *Atp5g3* and alcohol and obesity. The expression patterns of *Atp5g3* and each of its 12 partner genes/transcripts varied greatly in the same tissue. The correlation in expression levels between *Atp5g3* and each of these partners are different in tissues and genes. Transcriptome QTL mapping indicates that the *Atp5g3* is differentially regulated in hippocampus, cerebellum, and liver. However, there is currently no reported polymorphism in *Atp5g3* and its immediate up- and downstream among three inbred strains—C57BL/6J, DBA/2J, and BALB/cJ.

## Conclusions

The potential role of variants in *Atp5g3* in both alcoholism and metabolism warrants further investigation. In particular, the focus should be on immediate genetic regulation of *Atp5g3* expression and to adduce further evidence to support or refute potential causal links.

